# Editorial

**DOI:** 10.1017/ehs.2018.1

**Published:** 2019-04-23

**Authors:** Ruth Mace

**Affiliations:** Dept of Anthropology, UCL

## Abstract

**Figure:**
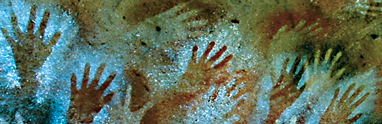

I am excited and somewhat daunted by the task of getting this journal off the ground. Editing a journal is like having a baby – you only decide to do it a second time when you have forgotten how hard it was the first time. It might be a crazy idea to open a new journal, when we are spoilt for choice where to send our work. Fortunately, 14 out of 15 reviewers approached by Cambridge University Press disagreed with that and thought that there was a need for a new, broad-based, high-quality, open access journal covering the full range of Evolutionary Human Sciences; so here it is.

I want to publish papers on anything to do with human evolution broadly defined, from human and primate phylogeny to physiology, genetics, demography, behaviour, cognition, language and culture. Evolutionary models are helping us understand classic topics in anthropology, like social organisation, kinship, warfare and witchcraft, in a new light. Evolutionary theory is informing psychology, medicine and public health, including why we harm others or ourselves. New species of hominin keep being unearthed. Collaborations between phylogeneticists, linguists and archaeologists are enhancing our understanding of our migrations and our cultural evolution. Advances in our ability to decode ancient and modern DNA are quite simply revolutionising our understanding of our own origins.

One reason I wanted this journal to exist was because, for someone like me working on human behavioural ecology and cultural evolution, there was always something not quite right about most of the journals on offer, however excellent they were. If you publish in social science journals there is little or no evolution, and if you publish in biology journals most of the work is on other species and humans are the outlier. The more specialist human behaviour journals don't necessarily cover the full range of human evolutionary studies. Nor are they open access. From the haven of my desk in London, with UCL's comprehensive online library at my fingertips, I was lucky enough not to have to worry too much about access, but having spent time working elsewhere, most recently in western China, the benefits to students and researchers of just being able to download something without any hassle became painfully obvious.

Cambridge University Press were astonished that the name *Evolutionary Human Sciences* was unclaimed. Why did such a journal not exist? Well, most of us know the history of evolutionary studies in the social sciences. Suffice it to say that the social sciences rejected evolutionary thinking *en masse*, and some rejected science altogether. However, the field of evolutionary social sciences began to create itself, without waiting to be invited to anybody else's party. A few anthropologists chose the path less trodden into evolutionary anthropology. Others entered the field from zoology, archaeology and psychology, a few from linguistics and philosophy (and some from economics, but most found it easier to stay put and make forays into the borderlands). The evolutionary study of human behaviour has been growing as a discipline in North America since the 1970s, but for a long time most of us in Europe were slightly isolated and just had to get on with it by ourselves. Diverse behavioural scientists with an interest in evolutionary approaches found each other, and gradually in Europe we accumulated a critical mass. So, many years after the same process had begun on the other side of the pond, we created our own institutions for evolutionary social scientists, of which I hope this journal will become one.

The more biological and physical areas of the evolutionary human sciences, like paleoanthropology and genetics, have deep roots and their own long-established societies, conferences and journals. We nonetheless encourage those of you researching in these fields to send your work to this journal too; and I especially urge you to consider sending us the more interdisciplinary studies that are emerging. Links between parts of genetics, archaeology and anthropology are proving astonishingly productive. Human evolutionary biology is a hotter topic than ever. New centres, new departments, new institutes, new academic societies and new conferences have opened up to keep up with it all. EHBEA is one such institution, that I am very happy to be associated with, which is affiliated to this journal. Indeed, if you join EHBEA you will get a significant discount on our publication charges, when they eventually come into effect.

Our field still faces plenty of challenges. The ethics of studies on human subjects are a matter of continual negotiation with the public. The replication crisis has hit evolutionary psychology particularly hard, but no area of our science can be immune from error. We are asked to examine our biases in science as in life (some unreplicated studies were just sexist or racist nonsense). We welcome open science and ask contributors to provide data and code where possible; and we offer the opportunity for registered reports. However, this is not as straightforward as it seems. Some non-experimental areas of the human sciences will be particularly hard to replicate in this formal way, and even controlled laboratory experiments are proving inconsistent – the human phenotype is just so damn variable and everything we do is context dependent!

Incidentally, reviewer number 15, who did not recommend CUP start this journal, agreed that it was a nice idea, but feared for the future of any new journal venturing into the competitive brew of academic publishing. Open access journals are, at least in part, a public good, and we all know what happens in public goods games. Even son number 2 commented “mum this journal thing sounds like a pretty altruistic venture”. It is sobering to remember that to play fair, you and your co-authors should, between you, review at least two papers for each one you write. Those experiments that showed that images of eyes on a computer screen were enough to make people donate to the public good have, unfortunately, not been replicated. There are, however, plenty of experiments showing that we care about our reputations, so please be a good science citizen (and fortunately the ScholarOne software keeps track of just how much reviewing everyone has been doing). Seriously though, I will need your support for the journal to flourish. I know you are too busy, but review our papers please (it need not take long). Send us your best work. And enjoy our final product.

We are sneaking onto the scene quietly and will publish any papers as soon as they are ready. Our formal launch will be at the 2019 EHBEA (European Human Behaviour and Evolution Association) conference in Toulouse in April, where you can meet the editors. We are starting with the relatively conventional formats of research articles, reviews, and registered reports. I am planning to add new formats in due course, and all suggestions are gratefully received. Don't hesitate to contact me on any matter and help us steer the journal in directions you want. Thank you in advance to all our wonderful editors and reviewers and authors for being part of this project. I am really looking forward to seeing this journal grow into something that you will hopefully all value.

